# Identification and Validation of Reference Genes and Their Impact on Normalized Gene Expression Studies across Cultivated and Wild *Cicer* Species

**DOI:** 10.1371/journal.pone.0148451

**Published:** 2016-02-10

**Authors:** Dumbala Srinivas Reddy, Pooja Bhatnagar-Mathur, Palakolanu Sudhakar Reddy, Katamreddy Sri Cindhuri, Adusumalli Sivaji Ganesh, Kiran Kumar Sharma

**Affiliations:** Genetic Transformation Laboratory, International Crops Research Institute for the Semi-Arid Tropics (ICRISAT), Patancheru-502324, Telangana, India; Chinese Academy of Sciences, CHINA

## Abstract

Quantitative Real-Time PCR (qPCR) is a preferred and reliable method for accurate quantification of gene expression to understand precise gene functions. A total of 25 candidate reference genes including traditional and new generation reference genes were selected and evaluated in a diverse set of chickpea samples. The samples used in this study included nine chickpea genotypes (*Cicer* spp.) comprising of cultivated and wild species, six abiotic stress treatments (drought, salinity, high vapor pressure deficit, abscisic acid, cold and heat shock), and five diverse tissues (leaf, root, flower, seedlings and seed). The geNorm, NormFinder and RefFinder algorithms used to identify stably expressed genes in four sample sets revealed stable expression of *UCP* and *G6PD* genes across genotypes, while *TIP41* and *CAC* were highly stable under abiotic stress conditions. While *PP2A* and *ABCT* genes were ranked as best for different tissues, *ABCT*, *UCP* and *CAC* were most stable across all samples. This study demonstrated the usefulness of new generation reference genes for more accurate qPCR based gene expression quantification in cultivated as well as wild chickpea species. Validation of the best reference genes was carried out by studying their impact on normalization of aquaporin genes *PIP1;4* and *TIP3;1*, in three contrasting chickpea genotypes under high vapor pressure deficit (VPD) treatment. The chickpea *TIP3;1* gene got significantly up regulated under high VPD conditions with higher relative expression in the drought susceptible genotype, confirming the suitability of the selected reference genes for expression analysis. This is the first comprehensive study on the stability of the new generation reference genes for qPCR studies in chickpea across species, different tissues and abiotic stresses.

## Introduction

Gene expression studies have become extremely important to obtain insights into gene function and to understand the molecular mechanisms. Among various techniques used for gene expression studies, quantitative Real-Time PCR (qPCR) has become the most important and reliable method owing to its accuracy and high-throughput analysis [1]. While appropriate applications of qPCR requires robust reference genes for accurate normalization, non-availability can result in experimental deviations or errors that inevitably occur during sample preparation leading to unreliable quantification of gene transcripts. Ideally, the endogenous genes selected as reference genes for qPCR for normalization of gene expression data should be expressed stably in all plant tissues under various experimental conditions [2]. Various housekeeping genes such as, glyceraldehydes-3-phosphate dehydrogenase (*GAPDH*), actin (*ACT*) tubulin (*TUB*) cyclophilin (*CYP*), elongation factor (*EF1*) and ribosomal RNA (*18S* and *28S rRNA*) have been widely used as reference genes in normalization of qPCR data. However, several studies have revealed that the expression levels of many commonly used housekeeping genes vary across tissues, treatments and species [1, 3–5]. Hence, emphasis has been on the identification of new generation reference genes that are stable under different experimental conditions [3, 6]. Since several studies have shown that no single universal gene has consistent expression under all experimental conditions, the evaluation of reference gene(s) under specific experimental conditions is essential for reliability of qPCR analysis [7, 8]. Moreover, several algorithms such as geNorm [9], NormFinder [10], BestKeeper [11] and comparative ∆Ct method [12] have been developed to evaluate the most stable reference gene(s) from set of candidate genes.

Studies on the evaluation of reference genes have been carried out in several plant species such as *Arabidopsis* [13], *Brassica juncea* [14], *Brassica napus* [15, 16], *Coffea* species [17], *Gossypium hirsutum* [18], *Oryza sativa* [19], *Solanum tuberosum* [20], *Solanum lycopersicum* [6], *Triticum aestivum* [21], *Vitis vinifera* [22], and *Zea mays* [23]. More recently reference gene validations have also been reported in a number of plants such as *Atropa belladonna* [24], *Caragana korshinskii* [25], *Panicum virgatum* [26], *Pennisetum glaucum* [27] *Phalaenopsis* [28] *Populus euphratica* [29] and *Saccharum officinarum* [30]. Among the leguminous crops, except for *Glycine max* [31] and *Arachis hypogaea* [1], very few reports are available on the evaluation of reference genes for qPCR studies in *Vicia faba* [32], in *Pisum sativum* [33] in *Lens culinaris* [34] and in *Cicer arietinum* [35]. Since, there has been a major emphasis on using biotechnological interventions including functional genomics and trans-genomics for various biotic and abiotic constraints in legumes, there is a need to evaluate species-specific reference genes under diverse environmental conditions.

Chickpea is an important food legume of the semi-arid tropical (SAT) regions of the world, known to be a *nutraceutical* (or health benefiting food) because of its high nutritional value [36]. Despite growing demand and high yield potential, chickpea yield is unstable and productivity is stagnant at unacceptably low levels due to constraints such as abiotic stresses (drought, heat, cold and high-salinity) and biotic stresses (*Ascochyta* blight, *Fusarium* wilt and pod borer). With chickpea genome sequencing allowing high-powered functional genomics studies to proceed [37], these can significantly accelerate molecular breeding efforts for the discovery and introgression of stress tolerance genes into cultivated germplasm. Nevertheless, for crop improvement in the post genomic era, there is a need to understand the function of genes in response to various stresses and during stages of growth and developmental, thereby necessitating gene expression profiling to identify the candidate genes.

Keeping this in view, we have evaluated a set of reference genes including traditional (commonly used) and new generation reference genes in a diverse set of biological samples of chickpea, including nine genotypes representing cultivated and wild species across primary, secondary and tertiary gene pools, plant tissues from various developmental stages, and six abiotic stress treatments (drought, salt, high vapor pressure deficit, abscisic acid, cold and heat shock). To identify the most stable reference gene(s) for normalization of qPCR data, this study evaluated 25 reference genes, including ATP-binding cassette transporter (*ABCT*), alcohol dehydrogenase class-3 (*ADH3*), calcium-dependent protein kinase 4 (*CDPK4*), clathrin adaptor complexes medium (*CAC*), cyclophilin (*CYP*), eukaryotic elongation factor 1-alpha (*ELF1a*), eukaryotic elongation factor 1-beta (*ELF1b*), galactose oxidase/kelch repeat superfamily protein (*FBOX*), glucose-6-phosphate dehydrogenase (*G6PD*), glyceraldehyde3-phosphate dehydrogenase (*GAPDH*), heat shock cognate protein 80 (*HSP80*), translation initiation factor IF-3 (*IF3*), eukaryotic initiation factor 4A-15 (*IF4a*), peroxin4 (*PEX4*), protein phosphatase2A subunit A3 (*PP2A*), pentatrico peptide repeat superfamily protein (*PPR*), s-adenosyl methionine decarboxylase (*SAMDM*), *SAND*-family protein (*SAND*), F-box protein SKIP16-like (*SKIP16*), *TIP41*-like protein (*TIP41*), un-characterized conserved protein UCP022280 (*UCP*), unknown protein (*UNK*), ubiquitin-protein ligase 7 (*UPL7*), vacuolar protein sorting-associated protein 53 homolog (*VPS*) and yellow leaf specific protein 8 (*YLS8*). The expression stability of these genes was evaluated across gene pools, different tissues and stress treatments using geNorm, NormFinder and RefFinder algorithms. Further, we have selected the best and least stable reference genes from the all samples set and validated by normalizing the expression levels of two aquaporin genes *PIP1;4* (Plasma membrane intrinsic protein) and *TIP3;1* (Tonoplast intrinsic protein) of chickpea. These two aquaporin genes express differentially in susceptible and tolerant genotypes of chickpea under different abiotic stress conditions (Unpublished data). The expression levels of the selected aquaporin genes under vapor pressure deficit (VPD) stress were tested in three chickpea genotypes with different tolerance levels.

## Materials and Methods

### Plant materials and stress treatments

Seeds of the chickpea cultivars and wild species were obtained from the mini core collection of the International Crop Research Institute for the Semi-Arid Tropics (ICRISAT), Hyderabad, India. Chickpea plants were grown in 8-inch pots containing 4.5 kg of black clay soil (Vertisol, 20% water holding capacity) under glasshouse conditions with 28/20°C day/night temperature. Nine chickpea genotype representations from cultivated genotypes and wild species across primary, secondary and tertiary gene pools (Table 1) were used for leaf tissue sample collection under normal growth conditions after 28 d from sowing. Besides, different tissue samples from seedlings of var. JG11 including leaf, flower, seed and roots were collected at different growth stages under normal growth conditions. For various abiotic stress treatments, 28-day-old vegetative stage plants of var. JG11 were used. Drought stress was imposed progressively and at the end of the dry-down cycle where normalized transpiration ratio (NTR) reached at 0.1 (10% of soil moisture remaining in the pot) [38], leaf samples were collected. Salinity stress imposition was done by completely saturating the potted plants with 150 mM NaCl, following collection of leaf samples after 24 h of treatment. For vapor pressure deficit (VPD) treatments, plants were shifted to growth chamber (31°C and 60% RH) one day before the VPD treatment for acclimatization, and subjected to a progressive VPD regime (0.67 kPa to 4.2 kPa) for 8 h [39] followed by the collection of leaf samples. Abscisic acid (ABA) stress was imposed on plants under glasshouse conditions by spraying the plants with 100 μM ABA solution and collection of leaf samples after 4 h. For cold (low temperature) and heat shock treatments, the plants were kept at 4°C and 40°C, respectively, and leaf samples collected after 4 h of treatment. For each sample, biological replicates were collected from three plants under the same experimental condition. The samples from both control and treated tissues of chickpea were immediately frozen in liquid nitrogen and stored at -80°C until RNA extraction.

**Table 1:** Details of the chickpea genotypes used for evaluation of candidate reference genes.

S. No.	Genotypes	Accession number	Remarks
1	*Cicer arietinum*	ICCV 93954	Cultivated desi type- JG11
2	*Cicer arietinum*	CDC Frontier	Cultivated Kabuli type
3	*Cicer reticulatum*	ICC 17121	Primary gene pool
4	*Cicer echinospermum*	ICC 17159	Secondary gene pool
5	*Cicer judaicum*	ICC 17192	Tertiary gene pool
6	*Cicer yamashitae*	ICC 17117	Tertiary gene pool
7	*Cicer pinnatifidum*	ICC 17126	Tertiary gene pool
8	*Cicer cuneatum*	Ec 600098	Tertiary gene pool
9	*Cicer bijugum*	ICC 17157	Tertiary gene pool

### Selection of candidate reference genes and primer design

The candidate genes were selected based on a review of studies in model plant *Arabidopsis* and other grain legumes such as soybean, chickpea and peanut followed by an *in silico* analysis using BLAST tools of the NCBI database. A total of 28 candidate reference genes that showed highly stable expression in previous studies including *CAC*, *FBOX*, *GAPC2*, *PEX4*, *PP2A*, *PPR*, *PTB1*, *SAMDM*, *SAND*, *TIP41*, *UCP*, *UNK*, *UPL7* and *YLS8* in *Arabidopsis* [3]; *ABCT*, *CDPK4*, *IF3* and *SKIP16* in soybean [40]; *ELF1a*, *GAPDH*, *HSP80* and *IF4a* in chickpea [35]; *ACT*, *ADH3*, *CYP*, *ELF1b* and *G6PD* in peanut [1] and the *VPS* gene from chickpea (unpublished) were selected for evaluation. To retrieve the orthologous mRNA and their corresponding DNA sequences in chickpea, mRNA sequences of the *Arabidopsis* genes, EST sequences of the chickpea, soybean and peanut were used to query databases of the National Center for Biotechnology Information (NCBI) using BLASTX. The retrieved mRNA sequences of the chickpea sequences were used to design PCR primers using Primer3 software [41] with GC content between 45 and 50%, primer length of 20–22 nucleotides, and an expected product size of 100–150 base pairs. Most of the primer pairs were designed from two adjacent exons, which were separated by an intron (Table 2).

**Table 2:** Details of the chickpea candidate reference genes and primer sequences used for qPCR analysis.

S. No.	Gene	Accession no *	Gene description	Homologue Accession no #	E-value	Primer sequence 5'-3' F/R	Amplicon length (bp)	Primers location@	PCR Efficiency
1	*ABCT*	XM_004505589	ATP-binding cassette transporter	XM_006591799	1e-161	TCACAGGTTGTGATGGAGTCTG	135	D	1.09
						CCTCAAATCTTGTTGGGGTGTC			
2	*ADH3*	XM_004493784	Alcohol dehydrogenase class-3-like	EG529529	6e-129	CGGATTATTGGCATAGACATCG	115	D	1.03
						CACTTATGACCTGCTGAATTGG			
3	*CDPK4*	XM_004496559	Calcium-dependent protein kinase 4-like	XR_136899	3e-125	GAACCTTCTCAAAGGCTCACTG	121	D	1.00
						CAAGATGCCACTCTCCACAACT			
4	*CAC*	XM_004511726	Clathrin adaptor complexes medium subunit family protein	AT5G46630	0.0	CATGGACTAGACCACCAATTCA	110	D	1.02
						AACAGTGTTGTACCCGCTCTTT			
5	*CYP*	XM_004500685	Cyclophilin -peptidyl-prolyl cis-trans isomerase-like	EE127717	7e-105	GGATGTTGTGAAGGAGATCGAG	133	S	0.93
						GAAGACTCAACGTCGCACAATC			
6	*ELF1a*	XM_004489495	Elongation factor 1-alpha	AJ004960	0.0	GTGGTTTTGAGGCTGGTATCTC	134	D	1.06
						GGCCTTTGAGTACTTGGGTGTA			
7	*ELF1b*	XM_004491150	Elongation factor 1-beta	EE126175	1e-40	GGTGATGAAACAGAGGAGGAGA	131	D	1.07
						ATGTCTGTCTCGTCATCCCAAG			
8	*FBOX*	XM_004491898	Galactose oxidase/ kelch repeat superfamily protein	AT5G15710	0.0	CACCACTCGGTTTGATGATG	148	S	1.02
						GTGCTGTAAAGTCCGATCCTTC			
9	*G6PD*	XM_004489522	Glucose-6-phosphate 1-dehydrogenase	EG030635	1e-108	ACAACGATACCAGGGTGTTACC	116	D	0.90
						TCTCCCATGATGCCTTTAACTC			
10	*GAPDH*	XM_004515773	Glyceraldehyde-3-phosphate dehydrogenase, cytosolic-like	AJ010224	0.0	GGCATTCTCGGATACACTGAAG	146	D	0.93
						TAGCCCAACTCGTTGTCATACC			
11	*HSP80*	XM_004491473	Heat shock cognate protein 80-like	GR406804	0.0	GGACTGAGCATTGATGAGGATG	148	S	0.94
						GTTCCTCGATCTCACACCTTTC			
12	*IF3*	XM_004507697	Translation initiation factor IF-3-like	NM_001255722	5e-93	CAGAAGAAGAAAAGGGATCAGC	119	D	0.94
						CGTGCAGCTTTCAAACGTACT			
13	*IF4a*	XM_004513380	Eukaryotic initiation factor 4A-15-like	FL512356	0.0	AGTCACTTCGGCCAGATTACAT	137	D	0.96
						AGCAGAGAAAACTCCCACTTGA			
14	*PEX4*	XM_004501123	Peroxin4	AT5G25760	5e-111	AGTATCCTCTGCAACCACCTCA	137	D	0.99
						ACAGACAGACTGCAGAGTCCAA			
15	*PP2A*	XM_004497052.	Protein phosphatase 2A subunit A3	AT1G13320	0.0	GCAGCATCAAAAGACAGAGTGC	117	D	0.98
						AACCAGACATGGTCGGATAGTC			
16	*PPR*	XM_004487860	Pentatrico peptide repeat (PPR) superfamily protein	AT5G55840	0.0	CTAAGGCATTGGAGTTGAGGAG	102	S	1.01
						TATCACCATCGGCACATAGACC			
17	*SAMDM*	XM_004502018	S-adenosyl-L-methionine-dependent methyl transferases superfamily protein	AT2G32170	0.0	GTGACCAACTTCGTCCTGTTTC	115	D	0.95
						TGGCTCGGATCACTGTAGACTT			
18	*SAND*	XM_004505939	SAND family protein	AT2G28390	0.0	CATGATAAAGGAATCGGACCAC	148	D	1.00
						CACGGTTGCATGTCTTTATTGC			
19	*SKIP16*	XM_004507288	F-box protein SKIP16-like	NM_001254106	0.0	GTCAGGTTCCATTGAAGGTTCC	149	D	1.03
						GGATAGCTGAGTCCCATAACGA			
20	*TIP41*	XM_004496854	Tonoplast intrinsic proteins -like protein	AT4G34270	2e-146	GTTGTACTTCGGGAGAGTTGCT	115	D	0.97
						GGAGCTTCTGGCTTATGATGCT			
21	*UCP*	XM_004505295	Uncharacterized conserved protein	AT4G26410	3e-82	TGGAGCCCAATTACAAAAGC	138	D	1.02
						TTTGAAGCCAAAGAGGCAAC			
22	*UNK*	XM_004499013	Unknown protein	AT4G33380	2e-118	CCTGATGGCATAGAGGATTCAG	139	D	0.93
						CAGCTGCACTATCTTTGTGGTG			
23	*UPL7*	XM_004494061	Ubiquitin-protein ligase 7	AT3G53090	0.0	GTCACAAGTTGTTCTCGTGCTC	147	D	0.92
						GTAGCAGGTTGAAGCTGATGGA			
24	*VPS*	XM_004486353	Vacuolar protein sorting-associated protein 53 homolog	—	0.0	GGAATTTCAGCGGATATTGGAG	123	D	0.97
						GGCGCAATTGTAGGTGTAATCT			
25	*YLS8*	XM_004497725	mRNA splicing factor, thioredoxin-like U5 snRNP	AT5G08290	4e-103	GTCTTGTTGTCATCCGTTTTGG	139	D	1.01
						TTAAAGTCAGGCACCTCTGTGA			
26	*PIP1;4^*	XM_004490905	Plasma membrane intrinsic proteins	—	—	TCATTGGATCTTCTGGGTGGGA	92	S	—
						TGGACTTAAAGGGAATGGCTCTG			
27	*TIP3;1^*	XM_004495430	Tonoplast intrinsic proteins	—	—	CCCGTTTGATGGAGCATGCA	95	S	—
						GGACCGACCCATAGATCCAA			

* GenBank accession numbers of the chickpea mRNA sequences used for primer designing

# GenBank accession numbers of the mRNA/EST used to search homologues in chickpea

@ Primers location on two exons (D), or on single exon (S)

^Aquaporin genes used for validation

### Genomic DNA isolation and PCR

The genomic DNA from leaves of chickpea variety JG11 was isolated using NucleoSpin Plant II DNA isolation kit (Macherey-Nagel, Duren, Germany). PCR reactions for all the candidate genes were performed using genomic DNA as template and gene-specific primers designed for qPCR. The PCR was performed in a total volume of 25 μl using 100 ng of genomic DNA, 200 nM of each primer, 1.5 mM MgCl_2_, 200 μM dNTP and 1 U *Taq* polymerase (Invitrogen, life technologies, NY, USA). PCR samples were amplified in an Eppendorf Thermal Cycler with an initial denaturation at 95°C for 5 min followed by 35 cycles of 95°C for 1 min, 62–64°C for 1 min and 72°C for 3 min, followed by a final extension at 72°C for 10 min, and PCR products tested by using 1% agarose gel electrophoresis.

### RNA extraction and cDNA synthesis

Total RNA was extracted from 100 mg tissue by using NucleoSpin RNA plant kit (Macherey-Nagel, Duren, Germany) including in-column DNAse1 treatment. The concentration and purity of all RNA samples was tested using NanoVue plus spectrophotometer (GE health care, USA) at 260/280 nm absorbance, selecting the ones that ranged from 1.8 to 2.0. Integrity of the RNA was tested by using 1.4% agarose gel electrophoresis with SYBR safe DNA gel stain (Invitrogen-life technologies, USA). The total RNA sample (2.5 μg) was reverse transcribed to cDNA using SuperScript^®^ III first strand cDNA synthesis kit (Invitrogen, Life Technologies, NY, USA) and oligodT primer in 25 μL reaction, according to manufacturer’s protocol. The cDNA preparations were diluted 12-times with nuclease-free water (Qiagen, Valencia, CA, USA) to use as template in qPCR analysis. To confirm the total absence of genomic DNA, cDNA was used as a template for PCR amplification using *ADH3* and *G6PD* primer pairs spanning an intron and PCR was performed as mentioned above.

### Quantitative real-time PCR analysis

All qPCRs were carried out in Realplex (Eppendorf, Germany) Real-Time PCR system using SYBR Green in 96 well optical reaction plates (Axygen, Union City, CA, USA) sealed with ultra-clear sealing film (Platemax). The PCR reaction was performed in a total volume of 10 μL containing 5 μl of 2X SensiFAST^TM^ SYBR No-ROX (Bioline, UK) mix, 400 nM of each primer, 1.0 μL of diluted cDNA and nuclease-free water to make up the final volume. The reaction conditions were set as 2 min at 95°C (polymerase activation); 40 cycles of 15 s at 95°C, 30 s at 62°C with fluorescent signal recording. At the end, a final step of 15 s at 95°C, 30 s at 58°C and fluorescence measurement at each 0.5°C variation from 58°C to 95°C in 20 min was included to obtain the melting curve. No-template controls were included for each primer combinations. Pooled and diluted cDNA sample was used in qPCR to check the specificity of all the primer pairs and verified by using 2% agarose gel electrophoresis with SYBR safe DNA gel stain (Invitrogen-life technologies, NY, USA) prior to sequencing the amplified products. The sequences of the PCR amplified products of each primer combination were compared with GenBank sequences using the BLASTN algorithm to check the PCR product specificity. For expression profiling, all the cDNA samples were tested in qPCR with each primer pair and three technical replicates performed for each cDNA sample of each biological replicate. The quantitative cycle (Cq) values were recorded using default settings of Real time PCR system where baseline was corrected automatically and threshold value was estimated by setting to noise band mode. Statistical analysis (Mean and CV) of the Cq values was carried out using Microsoft Excel spreadsheet 2010. The PCR efficiency of each primer pair was evaluated by the dilution series method using a pooled cDNA sample of the chickpea variety JG11. The 12-times diluted pooled cDNA sample was taken and 2-fold serial dilutions were carried out. Five serially diluted cDNA samples were used as templates to construct the standard curves of each primer pair, where PCR composition and conditions were same as above. Standard curves were constructed using the RealPlex (Eppendorf, Germany) real time PCR instrument software by using linear regression based on the quantitative cycle (Cq) values for all dilution points in a series. The correlation coefficients (*R*^2^) and slope values were obtained from the standard curve, and corresponding PCR amplification efficiencies (*E*) were calculated using the slope of the calibration curve according to the following equation: E = (10^−1/slope^-1).

### Data analysis

All experimental samples were divided into different sets based on their nature. The tissue set comprised of leaf, flower, root, seedling and seed of chickpea variety JG11 grown under normal growth conditions (five samples). The abiotic stress treatment set comprised of eight leaf samples of chickpea variety JG11 grown under drought, salt, high VPD, ABA, cold, heat shock, drought control and control. The genotypes sample set comprised of leaf sample of four cultivars and five wild species (total nine genotypes listed in Table 1). All the 20 samples (JG11 was a common control in all sample sets) were analyzed together as ‘all samples set’. The geNorm and NormFinder algorithms of genEX Professional software (MultiD Analyses AB, Sweden) were used to identify stably expressed gene(s) in a set of sample analyzed. The raw Cq values of each gene were corrected according to their respective PCR efficiencies before converting them into relative quantities, and the mean values of the biological replicates were taken as the input data for the geNorm and NormFinder analysis. The pair-wise variation analysis was carried out to identify the optimal number of genes required for normalization in each sample set by using geNorm of qBase plus software (ver: 2.4; Biogazelle, Belgium) [42]. RefFinder, a web-based (http://www.leonxie.com/referencegene.php) tool that integrates the currently available four major computational programs {geNorm [9], NormFinder [10], BestKeeper [11] and comparative ∆Ct method [12]} and calculates the geometric mean for the comprehensive ranking was used to calculate the comprehensive ranks.

### Reference gene validation by aquaporin gene expression studies under drought

Two aquaporin genes *PIP1;4* (Plasma membrane intrinsic protein) and *TIP3;1* (Tonoplast intrinsic protein) of chickpea were selected as target genes for quantification of gene expression levels under high vapor pressure deficit (VPD) treatment. Three chickpea varieties with contrasting drought tolerance viz. a susceptible variety ICC1882 and two tolerant varieties ICC4958 and JG11 were selected for reference gene validations. Experimental conditions and sample collection followed were same as mentioned above. Gene expression levels of *PIP1;4* and *TIP3;1* were normalized using the two most stable reference genes (*ABCT* and *UCP*) and two least stable genes (*CYP* and *SKIP16*) individually and in combination. Relative expressions of these two aquaporin genes in drought stressed leaf samples were estimated by comparing with expression levels of leaf sample collected from GH grown control plants of same variety using the REST [43] software.

## Results

### Primer specificity and PCR efficiencies

The candidate reference genes selected in this study represent different functional classes and gene families, including traditional and new generation reference genes based on reports in *Arabidopsis*, soybean, peanut and chickpea. Primers designed for 28 candidate reference genes were tested with cDNA of chickpea variety JG11. Since the primer pairs of *ACT*, *GAPC2* and *PTB1* genes yielded more than one band with cDNA as template, these genes were eliminated from further analysis. All the remaining 25 primer pairs except for *PP2A* (The *PP2A* forward primer was designed from the exon-exon junction region) yielded single PCR product of expected size with the genomic DNA (S1A Fig). The selected 25 candidate reference genes produced single band on agarose gel (S1B Fig) and a single peak in melting curve using the cDNA template (S2 Fig). The amplification products of cDNA of 25 primer pairs when sequenced and searched in GenBank (NCBI) using the BLASTN algorithm, showed matches with the retrieved mRNA sequences of the chickpea, thereby demonstrating the gene specificity of the primer pairs used for qPCR. The amplification efficiencies (*E*) of the candidate reference genes ranged from 0.90 to 1.09 (Table 2).

### Expression profiling of the reference genes

**Fig 1:**
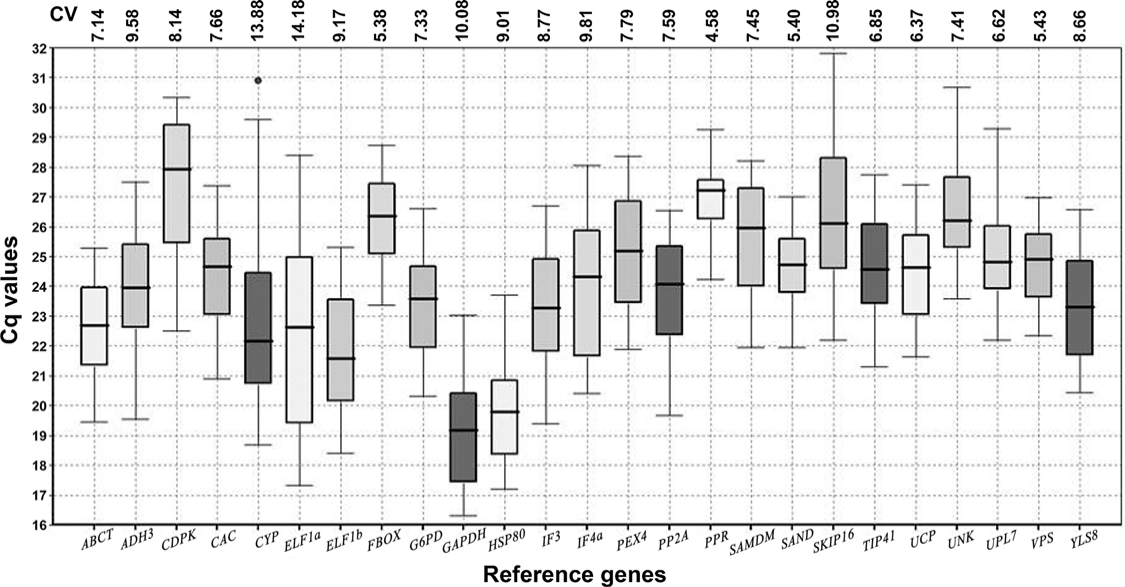
Expression levels of candidate reference genes across all samples. Lines across the boxes depict the medians. Boxes indicate the interquartile range. Whiskers represent 95% confidence intervals, black dot indicate the presence of outliers. Coefficient of variance (CV) of each gene among all samples is given in percentage.

A qPCR assay based on SYBR Green was used for transcript profiling of the 25 candidate reference genes across all 20 samples of chickpea including those from six abiotic stress conditions (drought, salt, high VPD, ABA, cold and heat shock), five different tissues (leaf, root, flower, seedlings and seed) and nine genotypes representing cultivated and wild species across the chickpea gene pools. To minimize the variability associated with qPCR analysis, all RNA samples were taken in equal concentration and quality for conversion to cDNA. The expression levels of the all candidate reference genes were individually determined in each of the samples as the quantification cycle value (Cq). A relatively wide range of Cq values across all 20 samples of chickpea was observed for the 25 reference genes, suggesting various levels of transcript abundance of the genes analyzed (Fig 1). The Cq values of all genes were in the range of 16.30–31.80 with a majority lying between 19 and 28 across all tested samples, with *GAPDH* exhibiting the lowest mean value (19.18) and *CDPK* the highest (27.55). Amongst all the tested genes, the *GAPDH*, *HSP80* and *ELF1b* showed higher expression where mean Cq values were below 22. The expression levels of *CDPK*, *PPR*, *SKIP16*, *UNK*, and *FBOX* were lower (mean Cq above 26), while the rest of the genes showed intermediate expression levels (mean Cq 22 to 26). The coefficient of variation (CV) values calculated for each reference gene indicated lower gene expression variation in *PPR* and *FBOX* (CV: 4.58 and 5.38, respectively) and the high variation in the expression of *ELF1a* (CV: 14.17) (Fig 1).

### Expression stability of the candidate reference genes

**Fig 2:**
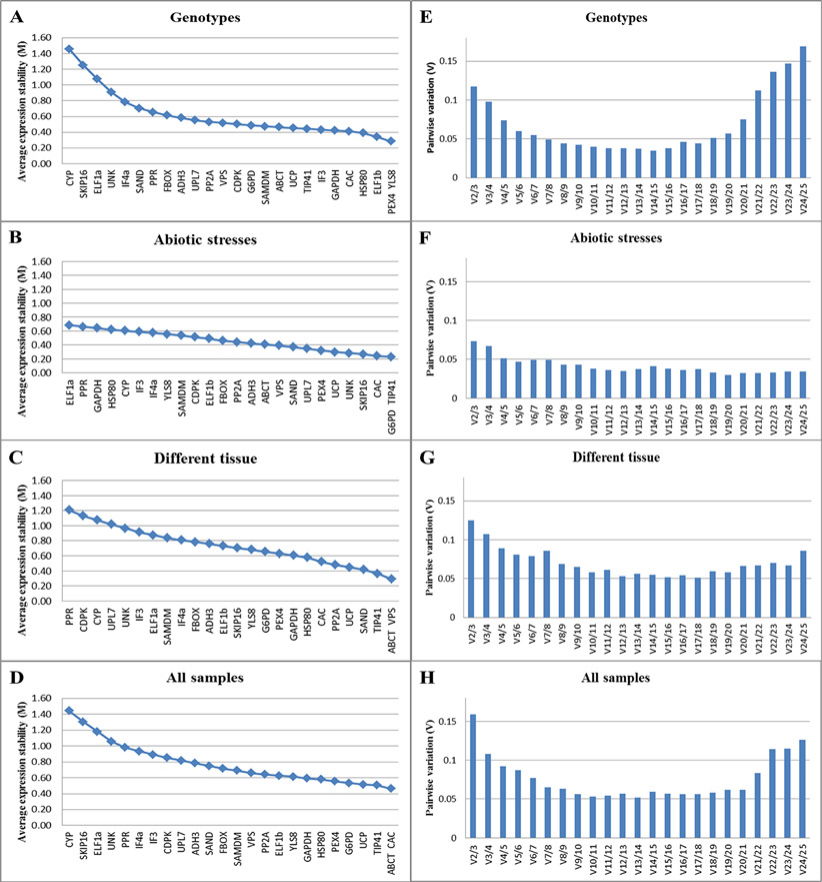
geNorm analysis. (A-D) Average expression stability and ranking of all 25 candidate reference genes: A lower value of average expression stability (M) indicates more stable expression. (E-H) Determination of the optimal number of reference genes for normalization by pairwise variation: The pairwise variation (Vn/Vn+1) was analyzed between normalization factors NFn and NFn+1 by geNorm algorithm to determine (V<0.15) the optimal number of reference genes. Error bars show standard deviation of relative expression of target genes in three biological replicates.

Since the stability value of the best reference gene or the best combination of genes may vary from one experimental set to another, the stability ranks of candidate genes in four sample sets was determined separately to identify the most suitable reference genes using geNorm and NormFinder algorithms. The geNorm program defines a stability measure (M) as the average pairwise variation between a gene and reference genes in a set of samples where genes with the lowest M values have the most stable expression. The lowest M value was observed for the pair *CAC*/*ABCT* (M = 0.46) corresponding to the most stable expression in all sample set, whereas the M values for *SKIP16* and *CYP* was considerably higher than the rest of the candidate genes. In tissue sample set, *VPS* and *ABCT* were most stable (M values 0.28) and *PPR*, *CDPK* were least stable (M values 1.20 and 1.12, respectively). Under abiotic stress conditions, while *TIP41* and *G6PD* were most stably expressed than all other candidate reference genes tested, *ELF1a* and *PPR* were unstable. The genes *YLS8* and *PEX4* were most stable in the tested genotypes across various gene pools while *CYP* and *SKIP16* were found to be least stable (Fig 2A–2D). Similarly, the stability ranks of candidate reference genes determined with NormFinder (Table 3**)** showed *UCP* as the most stable gene followed by *ABCT* and *G6PD* in the all sample set, whereas *CYP*, *SKIP16* and *ELF1a* genes were least stable among the all candidate reference genes tested. The *TIP41* expressed most stably under abiotic stress conditions followed by *CAC* and *PEX4*, whereas *ELF1a* and *PPR* remained least stable. Across genotypes and species, *UCP*, *G6PD* and *VPS* genes were identified as the most stable, whereas the *CYP* gene was identified as least stable followed by *SKIP16* and *ELF1a* genes. In the tissue sample set, *PP2A*, *CAC*, *ABCT* were identified as highly stable genes, whereas *PPR*, *CDPK*, *CYP* were among the least stable ones, according to NormFinder analysis.

**Table 3:** Gene expression stability ranks of all 25 candidate reference genes in four sample sets of chickpea calculated using geNorm (GN) and NormFinder (NF) algorithms.

Rank	Genotypes		Abiotic Stress		Different Tissue		All Samples	
	GN	NF	GN	NF	GN	NF	GN	NF
**1**	*PEX4*	*UCP*	*G6PD*	*TIP41*	*ABCT*	*PP2A*	*ABCT*	*UCP*
**2**	*YLS8*	*G6PD*	*TIP41*	*CAC*	*VPS*	*CAC*	*CAC*	*ABCT*
**3**	*ELF1b*	*VPS*	*CAC*	*PEX4*	*TIP41*	*ABCT*	*TIP41*	*G6PD*
**4**	*HSP80*	*SAMDM*	*SKIP16*	*ADH3*	*SAND*	*SKIP16*	*UCP*	*VPS*
**5**	*CAC*	*ABCT*	*UNK*	*UNK*	*UCP*	*TIP41*	*G6PD*	*SAMDM*
**6**	*GAPDH*	*CAC*	*UCP*	*SKIP16*	*PP2A*	*PEX4*	*PEX4*	*CAC*
**7**	*IF3*	*GAPDH*	*PEX4*	*UCP*	*CAC*	*VPS*	*HSP80*	*YLS8*
**8**	*TIP41*	*HSP80*	*UPL7*	*G6PD*	*HSP80*	*YLS8*	*GAPDH*	*HSP80*
**9**	*UCP*	*UPL7*	*SAND*	*UPL7*	*GAPDH*	*G6PD*	*YLS8*	*TIP41*
**10**	*ABCT*	*YLS8*	*VPS*	*ELF1b*	*PEX4*	*UCP*	*ELF1b*	*GAPDH*
**11**	*SAMDM*	*ELF1b*	*ABCT*	*PP2A*	*G6PD*	*SAND*	*PP2A*	*PEX4*
**12**	*G6PD*	*PEX4*	*ADH3*	*ABCT*	*YLS8*	*GAPDH*	*VPS*	*SAND*
**13**	*CDPK*	*CDPK*	*PP2A*	*SAMDM*	*SKIP16*	*HSP80*	*SAMDM*	*ELF1b*
**14**	*VPS*	*TIP41*	*FBOX*	*SAND*	*ELF1b*	*SAMDM*	*FBOX*	*FBOX*
**15**	*PP2A*	*SAND*	*ELF1b*	*CDPK*	*ADH3*	*ELF1b*	*SAND*	*PP2A*
**16**	*UPL7*	*IF3*	*CDPK*	*IF3*	*FBOX*	*ADH3*	*ADH3*	*UPL7*
**17**	*ADH3*	*PPR*	*SAMDM*	*VPS*	*IF4a*	*IF4a*	*UPL7*	*CDPK*
**18**	*FBOX*	*PP2A*	*YLS8*	*FBOX*	*SAMDM*	*FBOX*	*CDPK*	*ADH3*
**19**	*PPR*	*FBOX*	*IF4a*	*IF4a*	*ELF1a*	*IF3*	*IF3*	*IF4a*
**20**	*SAND*	*ADH3*	*IF3*	*YLS8*	*IF3*	*ELF1a*	*IF4a*	*PPR*
**21**	*IF4a*	*IF4a*	*CYP*	*HSP80*	*UNK*	*UNK*	*PPR*	*IF3*
**22**	*UNK*	*UNK*	*HSP80*	*CYP*	*UPL7*	*UPL7*	*UNK*	*UNK*
**23**	*ELF1a*	*ELF1a*	*GAPDH*	*GAPDH*	*CYP*	*CYP*	*ELF1a*	*ELF1a*
**24**	*SKIP16*	*SKIP16*	*PPR*	*PPR*	*CDPK*	*CDPK*	*SKIP16*	*SKIP16*
**25**	*CYP*	*CYP*	*ELF1a*	*ELF1a*	*PPR*	*PPR*	*CYP*	*CYP*

### RefFinder analysis

The rankings of candidate reference genes in all four-sample sets as re-determined using the RefFinder were highly consistent with the ones obtained by using geNorm and NormFinder. The RefFinder analysis revealed that while *UCP*, *G6PD*, *CAC* and *YLS8* reliably expressed in chickpea genotypes across species, *ABCT*, *UCP*, *CAC* and *G6PD* were most stable in all samples set. Similarly, while *PP2A*, *ABCT*, *VPS* and *CAC* genes were ranked as top four in differential tissue sample set, *TIP41*, *CAC*, *G6PD* and *PEX4* were observed to be highly stable under abiotic stress conditions. The comprehensive ranking revealed that the *CYP* gene was most unstable gene in all the sample sets, except under abiotic stresses. Various other genes such as *ELF1a* and *GAPDH* in abiotic stress, *CDPK* in differential tissues, *SKIP16* in genotypes across species and all samples were also found unreliable in this analysis (Table 4).

**Table 4:** Gene expression stability ranks of all 25 candidate reference genes in four sample sets of chickpea calculated using RefFinder.

Rank	Genotypes		Abiotic stress		Different tissue		All samples	
	Genes	Geomean of ranking values	Genes	Geomean of ranking values	Genes	Geomean of ranking values	Genes	Geomean of ranking values
**1**	*UCP*	3	*TIP41*	1.82	*PP2A*	2.85	*ABCT*	1.93
**2**	*G6PD*	5.26	*CAC*	3.03	*ABCT*	2.91	*UCP*	2.91
**3**	*CAC*	5.57	*G6PD*	4.6	*VPS*	4.28	*CAC*	3.46
**4**	*YLS8*	5.69	*PEX4*	5.54	*CAC*	4.53	*G6PD*	4.68
**5**	*PEX4*	5.7	*SKIP16*	5.57	*TIP41*	5.01	*VPS*	5.83
**6**	*VPS*	5.96	*SAND*	6.24	*UCP*	6.06	*TIP41*	5.9
**7**	*ABCT*	6.65	*UNK*	6.47	*SAND*	6.92	*HSP80*	8.43
**8**	*HSP80*	6.7	*UCP*	8.13	*PEX4*	8.22	*SAND*	8.57
**9**	*SAMDM*	6.85	*ADH3*	8.36	*SKIP16*	8.45	*FBOX*	9.53
**10**	*ELF1b*	8.44	*UPL7*	8.74	*YLS8*	9.5	*PEX4*	9.55
**11**	*FBOX*	8.73	*VPS*	9.35	*FBOX*	10.09	*PPR*	9.69
**12**	*UPL7*	8.97	*ABCT*	9.62	*GAPDH*	10.89	*SAMDM*	9.82
**13**	*GAPDH*	9.32	*PP2A*	9.62	*HSP80*	11.06	*YLS8*	9.89
**14**	*TIP41*	9.48	*FBOX*	11.76	*PPR*	11.18	*GAPDH*	10.47
**15**	*SAND*	10.47	*PPR*	12.89	*G6PD*	11.49	*UPL7*	12.15
**16**	*PPR*	12.52	*ELF1b*	13.31	*ELF1b*	12.75	*PP2A*	12.15
**17**	*CDPK*	12.74	*SAMDM*	15.36	*ADH3*	13.77	*ELF1b*	13.02
**18**	*IF3*	13.14	*CDPK*	15.74	*IF3*	13.78	*CDPK*	17.96
**19**	*PP2A*	17.14	*IF3*	18.38	*SAMDM*	17.06	*ADH3*	18.16
**20**	*ADH3*	18.93	*CYP*	18.69	*IF4a*	18.33	*IF3*	18.66
**21**	*UNK*	20.63	*IF4a*	18.99	*ELF1a*	20.22	*UNK*	19.65
**22**	*IF4a*	21.25	*YLS8*	19.95	*UPL7*	20.92	*IF4a*	20.22
**23**	*ELF1a*	23	*HSP80*	21.73	*UNK*	21.71	*ELF1a*	23.48
**24**	*SKIP16*	24.25	*GAPDH*	23.25	*CYP*	22.21	*SKIP16*	23.75
**25**	*CYP*	24.75	*ELF1a*	25	*CDPK*	24.25	*CYP*	24.75

### Optimal number of reference genes for normalization

The optimal number of reference genes required for accurate normalization was estimated using pairwise variation analysis of geNorm algorithm. Since pairwise variation analysis showed that V2/3 value (0.159) was higher than 0.15, an ideal normalization will require to include *CAC*, *ABCT* and *TIP41* as reference genes to normalize gene expression data in all samples set. Nevertheless, in the abiotic stressed sample set, genotype samples set and different tissue sample set, only two genes would be sufficient since the V2/3 values in these three sample sets was inferior to the 0.15 cut-off level. However, different experimental sets clearly required a different set of reference genes for normalization of gene expression, based on pair wise variation analysis viz. *G6PD* and *TIP41* genes for abiotic stresses sample set, and *YLS8* and *PEX4* genes for genotype sample set. The genes, *ABCT* and *VPSP* were required for normalization of gene expression data in different tissue sample set, where the V2/3 value was 0.125 (Fig 2E–2H).

### Validation of the selected reference genes

**Fig 3:**
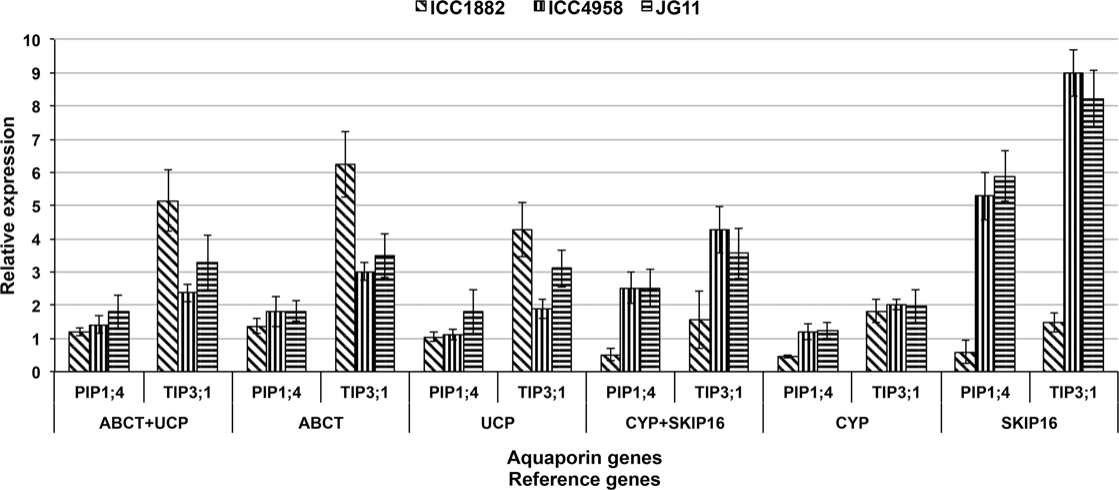
Relative quantification of aquaporin genes *PIP1;4* and *TIP3;1* to validate selected reference genes under drought stress conditions. Expression of *PIP1;4* and *TIP3;1* genes in high VPD treated chickpea leaf sample of three genotypes were relatively quantified by comparing with their control counterparts, with expression levels normalized with two most stable (*ABCT* and *UCP*) and least stable (*CYP* and *SKIP16*) reference genes.

To validate the selected reference genes in qPCR, the relative expression level of two aquaporin genes *PIP1;4* and *TIP3;1* were investigated in three chickpea varieties, ICC1882, ICC4958 and JG11 under VPD stress. For normalization of the relative expression level of two aquaporin genes, two most stable and two least stable reference genes as evaluated by RefFinder in all sample set were used (Table 4). This analysis revealed that the expression level of *PIP1;4* did not shown significant difference between drought susceptible and tolerant varieties, whereas *TIP3;1* shown difference, when normalized with *ABCT* and *UCP* reference genes individually or in combination. The relative expression levels of the two aquaporin genes were did not followed the above pattern, when normalized using *CYP* and *SKIP16* as the two most unstable reference genes, compared to the expression levels obtained after normalization with *TIP41* and *CAC* genes. Moreover, both genes *PIP1;4 TIP3;1* did not shown significant up regulation in susceptible variety ICC1882, when relative expression levels were normalized with *CYP* and *SKIP16* genes individually or in combination (Fig 3).

## Discussion

Microarray and next generation RNA sequencing technology-based studies has indicated that transcription levels vary between species, between ploidy levels and between tissue types [44]. The qPCR has become the first choice for accurate quantification of gene expression profiles of selected genes in diverse biological samples due to its sensitivity, accuracy and high throughput [45]. Nevertheless, stably expressing internal reference gene(s) are required for accurate gene expression. However, based on the previous reports, no single gene expresses stably across the experimental conditions [7, 8]. Hence making it, critical to identify a set of stable reference genes that show stable expression in distinct biological samples: across different genotypes, tissue, growth stages and under varied experimental conditions.

Genome sequence for both desi and Kabuli type chickpea is now available to researchers and functional genomics will be the major focus area in chickpea crop improvement programs [46, 37]. Since, qPCR will be a very useful tool in the identification of candidate genes that controls major traits by studying their expression profiles, it is critical to develop a reference gene tool-kit with stably expressing internal genes for transcript normalization under specific experimental conditions. While, a previous report has evaluated 12 commonly used reference genes in a desi type chickpea var. ICC 4958 [35], the present study evaluated the expression stability of 25 candidate reference genes including new generation candidate reference genes. Moreover, assessing these genes in diverse tissue samples including, genotypes of chickpea representing both cultivated and wild species across gene pools, different tissues/developmental stages and abiotic stress conditions, ensures the robustness of our results owing to a wide range of variability covered in our samples.

The expression profiling showed that all the 25 candidate reference genes varied significantly across the 20 samples tested. These results indicated that the most stable gene(s) among all the tested genes must be determined statistically. The stability value of the most suitable reference gene or the best combination of genes may vary from one experimental set to another, and for that reason the choice for reference genes for optimal normalization was made from a set of candidate genes for each experiment and for all of them together. To identify most stable reference gene under different experimental conditions, we grouped the diverse chickpea tissue samples into four sample sets and validated the 25 candidate reference genes using geNorm and NormFinder, two distinct statistical algorithms. Although, several studies have reported differences between the outputs of geNorm and NormFinder [8, 47, 48], we observed a high correlation between these rankings. In the present study, geNorm or NormFinder analysis in all four sample sets revealed a high degree of similarity. From the top 10 stable genes identified, 6 to 8 genes were almost same, with slight changes in their ranking orders. Interestingly, three most unstable genes remained same in all sample sets with both the algorithms (Table 3). To determine the comprehensive ranking of candidate reference genes in a sample set, we also used RefFinder that considered together the results of the four algorithms (geNorm, NormFinder, bestkeeper and deltCt method). Based on the stability rankings, different candidate reference genes have been proposed for their best suitability as reference genes depending on the experimental conditions. This being an important consideration would imply that the reference genes used for biotic or abiotic stress studies in chickpea might not be suitable for gene expression studies across genotypes and species.

Interestingly, new generation candidate reference genes selected for the current study performed better than the traditionally employed reference genes. In particular, *CAC* (*Arabidopsis* homologue: AT5G46630) was identified among the top four reference genes in all four-sample sets, indicating that *CAC* transcript levels were stable in chickpea under different experimental conditions tested. In this study, the *CAC* gene was selected as a candidate reference gene based on its highly stable expression in the *Arabidopsis* [3]. Besides, *CAC* has been reported to be the best reference gene for normalization in tomato [6], drought stressed roots and genotypes of coffee spp. [8, 17], during fruit development in cotton [18], across total developmental stages and cultivars in mustard [14], and at various developmental stages in bamboo [49]. Based on the present and previous studies, it can be concluded that the *CAC* gene is stably expressed in different experimental conditions.

The uncharacterized conserved protein UCP022280 (*UCP*, *Arabidopsis* homologue: AT4G26410) was most stably expressed in different genotypes of chickpea and ranked second in all sample set. Previously, *UCP* gene was identified as stably expressed gene in *Arabidopsis* microarray analysis [3], and different experimental conditions [50]. Interestingly, *UCP* gene expression has reportedly been moderately stable in *Arabidopsis* under metal stress [4] and buckwheat [51]. In contrast, the expression of *UCP* gene was most unstable in vegetative tissues and maturing embryos of rapeseed [52]. An ATP-binding cassette transporter (*ABCT*) gene known to play a variety of cellular roles such as auxin transport was also selected for validation in the present study based on the microarray and qPCR validation in soybean, where *ABCT* has been reported as most suitable for gene expression normalization [40]. In the present study, *ABCT* gene was most stable when all samples were analyzed together and second most stable gene in differential tissue sample set, which is in agreement with the study in soybean where the *ABCT* gene was expressed stably under various abiotic stress conditions [53]. Besides soybean, this study is the first to report *ABCT* as the stable reference gene in crop species. Similarly, the TIP41-like family protein (*TIP41*; Tonoplast Intrinsic Proteins like protein; At4g34270) coding gene that was selected based on the *Arabidopsis* microarray data [3] emerged as most stable gene under abiotic stress conditions, whereas ranked fifth in different tissues and sixth in all sample set. These observations are in accordance with previous studies where *TIP41* gene stably expressed under different abiotic conditions in desert shrub [54] and Chinese celery [55]. *TIP41* gene also showed stable and similar expression regulation across *Brassica* species regardless of experimental conditions [14, 16, 52] and has been reported to be one of the most stable reference gene in many other plant species including soybean [56], tomato [6], buckwheat [51], peanut [57], common hop [58], cucumber [59], bamboo [49], Chinese pear [60] and olive tree [61].

Since our previous studies indicated stable expression of glucose-6-phosphate dehydrogenase (*G6PD)* gene under different experimental conditions in peanut [1], this was selected as one of the candidate in the present study. Similar to peanut, *G6PD* gene ranked in top four most stably expressed genes across different chickpea genotypes under abiotic stress conditions and all samples set. However, these results are in contrast to the findings in soybean where *G6PD* gene expression was least stable under developmental stages and different photoperiodic treatments [56], under cadmium stress [62], and also varied most in all tested experimental conditions [63]. The vacuolar protein sorting-associated protein 53 homolog (*VPS*) gene selected as candidate reference gene in the present study has not been previously explored as reference gene for qPCR analysis, it was identified as low copy gene in the legumes (unpublished data) and subsequently cloned. Interestingly, *VPS* gene expressed stably in different tissues and showed moderate stability across all samples and genotypes, ranking 5^th^ and 6^th^ respectively, indicating its potential for inclusion as reference gene in expression studies in other crop species.

The expression of *CYP*, *ELF1a* and *IF4a* genes were highly variable among the genes evaluated under different experimental conditions. The *CYP* gene was least stable in all samples set, across genotypes and different tissues, and showed high variability under abiotic stress. These observations were intriguing since this was in contrast to our previous studies in peanut, where *CYP* gene expressed most stably in vegetative stages and under abiotic stress conditions [1], and various tissues of soybean [56] and common bean [32]. Similarly, in this study *ELF1a* gene was found least stable under abiotic stress conditions and showed highly variable expression in other three sample sets, whereas *IF4a* genes was highly variable in all the four sample sets. These observations are in contrast with the previous study in chickpea [35] where *ELF1a* and *IF4a* genes were reported among the top three stably expressed genes in all three experimental conditions. Besides, the other two genes *GAPDH* and *HSP80* that were previously reported to have stable expression [35], showed high variability under abiotic stress, but were moderately stable in other three sample sets. These differences justify the evaluation of this new set of reference genes indicating that, these new generation reference genes performed better than the conventional reference genes.

According to Vandesompele et al. [9] and as per the MIQE (Minimum Information for Publication of Quantitative Real-Time PCR Experiments) guidelines [45], two or more reference genes were required for accurate normalization of gene expression data. Several other reports also suggested that the application of more than one reference gene could lead to more reliable data in qPCR assays [18, 64]. The geNorm proposed 0.15 as the threshold of pairwise variation values, below which the inclusion of an additional reference gene is not required. In this study, three out of four sample sets (genotypes, different tissue and abiotic stress sample sets) showed pairwise variation V2/3 values below 0.15, thereby indicating that the two reference genes were enough for normalization under these experimental conditions, whereas for all sample set three reference genes were required for normalization. These high pairwise variation values in all sample set may have resulted due to a highly diverse sample set, i.e., highly diverse samples might have significant differences in gene transcription including those of the candidate reference genes and were likely related to the developmental and metabolic properties of each distinct tissue.

To validate the selected candidate reference genes based on RefFinder ranking, expression of two chickpea aquaporin genes (*PIP1;4* and *TIP3;1*) that belong to two different aquaporin sub-families (unpublished data) were assessed. Plant aquaporin proteins play major role in water transport through cell membranes and these genes are expressed differentially in different tissues that are also altered under different abiotic stresses in higher plants. The chickpea *TIP3;1* gene was significantly up regulated under high VPD conditions in leaf tissues of three chickpea genotypes with relatively higher expression in susceptible genotype ICC1882, when normalized with *ABCT* and *UCP* reference genes. These results were in accordance with our previous study in sorghum [65] and in chickpea (unpublished data), where up-regulation of *TIP* genes in leaf tissues was reported under different abiotic stress conditions, but the normalization was obscured when the least stable reference gene(s) (*CYP* and *SKIP16*) were used. The validation results clearly indicated the demerits of using unstable reference gene(s) for normalization and confirmed the reference gene stability of the selected candidate genes.

## Conclusions

The new generation candidate reference genes (*CAC*, *UCP*, *ABCT*, *G6PD*, *VPS* and *TIP41*) selected for the present study performed significantly better than the traditionally employed reference genes that were stably expressed across different experimental conditions. However, the *CYP* and *ELF1a* genes that were most unstable should be avoided for use as reference genes in gene expression studies in chickpea. Pairwise variation analysis indicated that two genes are enough for normalization of gene expression data in different sample sets except when all samples were analyzed together, where three genes are required. The validation of selected reference gene by normalizing aquaporin gene expression data further confirmed the stability of the *ABCT* and *UCP* as reference genes under high VPD conditions. These results provide information that can ensure more accurate qPCR based gene expression quantification towards chickpea functional genomics.

## Supporting Information

S1 Fig Amplification of specific PCR products: with genomic DNA (A) and cDNA (B) as templates using gene-specific primers for each candidate reference gene tested in the study. 1 to 25 indicates the loading order of the candidate reference genes as mentioned in Table 2, M- DNA size marker. All primer pairs except *CYP*, *FBOX*, *HSP80* and *PPR* amplified a larger size PCR product with DNA template as compared to cDNA template, indicating the position of primer pairs spanning at least one intron.
(TIF)

S2 Fig Melt curves of all 25 candidate reference genes evaluated in this study. (TIF)

## References

[1] Reddy DS, Bhatnagar-Mathur P, Cindhuri KS, Sharma KK. Evaluation and validation of reference genes for normalization of quantitative real-time PCR based gene expression studies in peanut. PloS One. 2013; 8: e78555 10.1371/journal.pone.0078555 24167633 PMC3805511

[2] Hong SY, Seo PJ, Yang MS, Xiang F, Park CM. Exploring valid reference genes for gene expression studies in *Brachypodium distachyon* by real-time PCR. BMC Plant Biol. 2008; 8: 112–122. 10.1186/1471-2229-8-112 18992143 PMC2588586

[3] Czechowski T, Stitt M, Altmann T, Udvardi MK, Scheible WR. Genome-wide identification and testing of superior reference genes for transcript normalization in *Arabidopsis*. Plant Physiol. 2005; 139: 5–17. 16166256 10.1104/pp.105.063743 PMC1203353

[4] Remans T, Smeets K, Opdenakker K, Mathijsen D, Vangronsveld J, Cuypers A. Normalisation of real-time RT-PCR gene expression measurements in *Arabidopsis thaliana* exposed to increased metal concentrations. Planta. 2008; 227: 1343–1349. 10.1007/s00425-008-0706-4 18273637

[5] Silberberg G, Baruch K, Navon R. Detection of stable reference genes for real-time PCR analysis in schizophrenia and bipolar disorder. Anal Biochem. 2009; 391: 91–97. 10.1016/j.ab.2009.05.026 19464249

[6] Exposito-Rodriguez M, Borges AA, Borges-Perez A, Perez JA. Selection of internal control genes for quantitative real-time RT-PCR studies during tomato development process. BMC Plant Biol. 2008; 8: 131–142. 10.1186/1471-2229-8-131 19102748 PMC2629474

[7] Olsvik PA, Softeland L, Lie KK. Selection of reference genes for qRT-PCR examination of wild populations of Atlantic cod *Gadus morhua*. BMC Res Notes. 2008; 1: 47 10.1186/1756-0500-1-47 18710500 PMC2527504

[8] Cruz F, Kalaoun S, Nobile P, Colombo C, Almeida J, Barros LM, et al Evaluation of coffee reference genes for relative expression studies by quantitative real-time RT-PCR. Mol Breed. 2009; 23: 607–616.

[9] Vandesompele J, De Preter K, Pattyn F, Poppe B, Van Roy N, De Paepe A, et al Accurate normalization of real-time quantitative RT-PCR data by geometric averaging of multiple internal control genes. Genome Biol. 2002; 3: research0034.1–0034.11. 12184808 10.1186/gb-2002-3-7-research0034 PMC126239

[10] Andersen CL, Jensen JL, Orntoft TF. Normalization of real-time quantitative reverse transcription-PCR data: a model-based variance estimation approach to identify genes suited for normalization, applied to bladder and colon cancer data sets. Cancer Res. 2004; 64: 5245–5250. 15289330 10.1158/0008-5472.CAN-04-0496

[11] Pfaffl MW, Tichopad A, Prgomet C, Neuvians TP. Determination of stable housekeeping genes, differentially regulated target genes and sample integrity: BestKeeper—Excel-based tool using pair-wise correlations. Biotechnol Lett. 2004; 26: 509–515. 15127793 10.1023/b:bile.0000019559.84305.47

[12] Silver N, Best S, Jiang J, Thein SL. Selection of housekeeping genes for gene expression studies in human reticulocytes using real-time PCR. BMC Mol Biol. 2006; 7: 33 17026756 10.1186/1471-2199-7-33 PMC1609175

[13] Hong SM, Bahn SC, Lyu A, Jung HS, Ahn JH. Identification and testing of superior reference genes for a starting pool of transcript normalization in *Arabidopsis*. Plant Cell Physiol. 2010; 51: 1694–1706. 10.1093/pcp/pcq128 20798276

[14] Chandna R, Augustine R, Bisht NC. Evaluation of candidate reference genes for gene expression normalization in *Brassica juncea* using real time quantitative RT-PCR. PloS One 2012; 7: e36918 10.1371/journal.pone.0036918 22606308 PMC3350508

[15] Yang H, Liu J, Huang S, Guo T, Deng L, Hua W. Selection and evaluation of novel reference genes for quantitative reverse transcription PCR (qRT-PCR) based on genome and transcriptome data in *Brassica napus* L. Gene. 2014; 538: 113–122. 10.1016/j.gene.2013.12.057 24406618

[16] Wang Z, Chen Y, Fang H, Shi H, Chen K, Zhang Z, et al Selection of reference genes for quantitative reverse-transcription polymerase chain reaction normalization in *Brassica napus* under various stress conditions. Mol Genet Genomics. 2014; 289: 1023–1035. 10.1007/s00438-014-0853-1 24770781

[17] Goulao LF, Fortunato AS, Ramalho JC. Selection of reference genes for normalizing Quantitative Real-Time PCR gene expression data with multiple variables in *Coffea* spp. Plant Mol Biol Rep. 2012; 30: 741–759.

[18] Artico S, Nardeli SM, Brilhante O, Grossi-de-Sa MF, Alves-Ferreira M. Identification and evaluation of new reference genes in *Gossypium hirsutum* for accurate normalization of real-time quantitative RT-PCR data. BMC Plant Biol. 2010; 10: 49 10.1186/1471-2229-10-49 20302670 PMC2923523

[19] Jain M, Nijhawan A, Tyagi AK, Khurana JP. Validation of housekeeping genes as internal control for studying gene expression in rice by quantitative real-time PCR. Biochem Biophys Res Comm. 2006; 345: 646–651. 16690022 10.1016/j.bbrc.2006.04.140

[20] Nicot N, Hausman JF, Hoffmann L, Evers D. Housekeeping gene selection for real-time RT-PCR normalization in potato during biotic and abiotic stress. J Exp Bot. 2005; 56: 2907–2914. 16188960 10.1093/jxb/eri285

[21] Long XY, Wang JR, Ouellet T, Rocheleau H, Wei YM, Pu ZE, et al Genome-wide identification and evaluation of novel internal control genes for Q-PCR based transcript normalization in wheat. Plant Mol Biol. 2010; 74: 307–311. 10.1007/s11103-010-9666-8 20658259

[22] Reid KE, Olsson N, Schlosser J, Peng F, Lund ST. An optimized grapevine RNA isolation procedure and statistical determination of reference genes for real-time RT-PCR during berry development. BMC Plant Biol. 2006; 6: 27 17105665 10.1186/1471-2229-6-27 PMC1654153

[23] Manoli A, Sturaro A, Trevisan S, Quaggiotti S, Nonis A. Evaluation of candidate reference genes for qPCR in maize. J Plant Physiol. 2012; 169: 807–815. 10.1016/j.jplph.2012.01.019 22459324

[24] Li JD, Chen M, Qiu F, Qin B, Liu W, Wu N, et al Reference gene selection for gene expression studies using quantitative real-time PCR normalization in *Atropa belladonna*. Plant Mol Biol Rep. 2014; 32: 1002–1014.

[25] Yang Q, Yin J, Li G, Qi L, Yang F, Wang R, et al Reference gene selection for qRT-PCR in *Caragana korshinskii* Kom. under different stress conditions. Mol Biol Rep. 2014; 41: 2325–2334. 10.1007/s11033-014-3086-9 24452712

[26] Gimeno J, Eattock N, Van Deynze A, Blumwald E. Selection and validation of reference genes for gene expression analysis in switchgrass (*Panicum virgatum*) using quantitative real-time RT-PCR. PloS One. 2014; 9: e91474 10.1371/journal.pone.0091474 24621568 PMC3951385

[27] Reddy PS, Reddy DS, Bhatnagar-Mathur P, Sharma KK, Vadez V. Cloning and validation of reference genes for normalization of gene expression studies in pearl millet [*Pennisetum glaucum* (L.) R. Br.] by quantitative real-time PCR. Plant Gene. 2015; 1: 35–42.

[28] Yuan XY, Jiang SH, Wang MF, Ma J, Zhang XY, Cui B. Evaluation of internal control for gene expression in *Phalaenopsis* by quantitative real-time PCR. Appl Biochem Biotechnol. 2014; 173: 1431–1445. 10.1007/s12010-014-0951-x 24811734

[29] Wang HL, Chen J, Tian Q, Wang S, Xia X, Yin W. Identification and validation of reference genes for *Populus euphratica* gene expression analysis during abiotic stresses by quantitative real-time PCR. Physiol Plant. 2014; 152: 529–545. 10.1111/ppl.12206 24720378

[30] Ling H, Wu Q, Guo J, Xu L, Que Y. Comprehensive selection of reference genes for gene expression normalization in sugarcane by real time quantitative RT-PCR. PloS One. 2014; 9: e97469 10.1371/journal.pone.0097469 24823940 PMC4019594

[31] Nakayama TJ, Rodrigues FA, Neumaier N, Marcelino-Guimaraes FC, Farias JR, de Oliveira MC, et al Reference genes for quantitative real-time polymerase chain reaction studies in soybean plants under hypoxic conditions. Genet Mol Res. 2014; 13: 860–871. 10.4238/2014.February.13.4 24615050

[32] Gutierrez N, Gimenez MJ, Palomino C, Avila CM. Assessment of candidate reference genes for expression studies in *Vicia faba* L. by real-time quantitative PCR. Mol Breeding. 2011; 28: 13–24.

[33] Die JV, Roman B, Nadal S, Gonzalez-Verdejo CI. Evaluation of candidate reference genes for expression studies in *Pisum sativum* under different experimental conditions. Planta. 2010; 232: 145–153. 10.1007/s00425-010-1158-1 20379832

[34] Saha GC, Vandemark GJ. Stability of expression of reference genes among different lentil (*Lens culinaris*) genotypes subjected to cold stress, white mold disease, and aphanomyces root rot. Plant Mol Biol Rep. 2013; 31: 1109–1115.

[35] Garg R, Sahoo A, Tyagi AK, Jain M. Validation of internal control genes for quantitative gene expression studies in chickpea (*Cicer arietinum* L.). Biochem Biophys Res Comm. 2010; 396: 283–288. 10.1016/j.bbrc.2010.04.079 20399753

[36] Jukanti AK, Gaur PM, Gowda CL, Chibbar RN. Nutritional quality and health benefits of chickpea (*Cicer arietinum* L.): a review. Br J Nutr. 2012; 108: S11–26. 10.1017/S0007114512000797 22916806

[37] Varshney RK, Song C, Saxena RK, Azam S, Yu S, Sharpe AG, et al Draft genome sequence of chickpea (*Cicer arietinum*) provides a resource for trait improvement. Nat Biotechnol. 2013; 31: 240–246. 10.1038/nbt.2491 23354103

[38] Vadez V, Sinclair TR. Leaf ureide degradation and N-2 fixation tolerance to water deficit in soybean. J Exp Bot. 2001; 52: 153–159. 11181724

[39] Zaman-Allah M, Jenkinson DM, Vadez V. Chickpea genotypes contrasting for seed yield under terminal drought stress in the field differ for traits related to the control of water use. Funct Plant Biol. 2011; 38: 270–281. 10.1071/FP10244 32480883

[40] Libault M, Thibivilliers S, Bilgin DD, Radwan O, Benitez M, Clough SJ, et al Identification of four soybean reference genes for gene expression normalization. Plant Genome. 2008; 1: 44–54.

[41] Untergasser A, Cutcutache I, Koressaar T, Ye J, Faircloth BC, Remm M, et al Primer3-new capabilities and interfaces. Nucleic Acids Res. 2012; 40: e115 22730293 10.1093/nar/gks596 PMC3424584

[42] Hellemans J, Mortier G, De Paepe A, Speleman F, Vandesompele J. qBase relative quantification framework and software for management and automated analysis of real-time quantitative PCR data. Genome Biol. 2007; 8: R19 17291332 10.1186/gb-2007-8-2-r19 PMC1852402

[43] Pfaffl MW, Horgan GW, Dempfle L. Relative expression software tool (REST) for group-wise comparison and statistical analysis of relative expression results in real-time PCR. Nucleic Acids Res. 2002; 30: e36 11972351 10.1093/nar/30.9.e36 PMC113859

[44] Wang H, Chen S, Jiang J, Zhang F, Chen F. Reference gene selection for cross-species and cross-ploidy level comparisons in *Chrysanthemum* spp. Sci Rep. 2015; 5: 8094 10.1038/srep08094 25627791 PMC4308696

[45] Bustin SA, Benes V, Garson JA, Hellemans J, Huggett J, Kubista M, et al The MIQE guidelines: minimum information for publication of quantitative real-time PCR experiments. Clin Chem. 2009; 55: 611–622. 10.1373/clinchem.2008.112797 19246619

[46] Jain M, Misra G, Patel RK, Priya P, Jhanwar S, Khan AW, et al A draft genome sequence of the pulse crop chickpea (*Cicer arietinum* L.). The Plant J. 2013; 74: 715–729. 10.1111/tpj.12173 23489434

[47] Ahn K, Huh JW, Park SJ, Kim DS, Ha HS, Kim YJ, et al Selection of internal reference genes for SYBR green qRT-PCR studies of rhesus monkey (*Macaca mulatta*) tissues. BMC Mol Biol. 2008; 9: 78 10.1186/1471-2199-9-78 18782457 PMC2561044

[48] Tong Z, Gao Z, Wang F, Zhou J, Zhang Z. Selection of reliable reference genes for gene expression studies in peach using real-time PCR. BMC Mol Biol. 2009; 10: 71 10.1186/1471-2199-10-71 19619301 PMC3224724

[49] Fan C, Ma J, Guo Q, Li X, Wang H, Lu M. Selection of reference genes for quantitative real-time PCR in bamboo (*Phyllostachys edulis*). PloS One. 2013; 8: e56573 10.1371/journal.pone.0056573 23437174 PMC3577859

[50] Berr A, McCallum EJ, Menard R, Meyer D, Fuchs J, Dong A, et al *Arabidopsis* SET DOMAIN GROUP2 is required for H3K4 trimethylation and is crucial for both sporophyte and gametophyte development. Plant Cell. 2010; 22: 3232–3248. 10.1105/tpc.110.079962 21037105 PMC2990135

[51] Demidenko NV, Logacheva MD, Penin AA. Selection and validation of reference genes for quantitative real-time PCR in buckwheat (*Fagopyrum esculentum*) based on transcriptome sequence data. PloS One. 2011; 6: e19434 10.1371/journal.pone.0019434 21589908 PMC3093374

[52] Chen X, Truksa M, Shah S, Weselake RJ. A survey of quantitative real-time polymerase chain reaction internal reference genes for expression studies in *Brassica napus*. Anal Biochem. 2010; 405: 138–140. 10.1016/j.ab.2010.05.032 20522329

[53] Le DT, Aldrich DL, Valliyodan B, Watanabe Y, Van Ha C, Nishiyama R, et al Evaluation of candidate reference genes for normalization of quantitative RT-PCR in soybean tissues under various abiotic stress conditions. PloS One. 2012; 7: e46487 10.1371/journal.pone.0046487 23029532 PMC3460875

[54] Zhu J, Zhang L, Li W, Han S, Yang W, Qi L. Reference gene selection for quantitative real-time PCR normalization in *Caragana intermedia* under different abiotic stress conditions. PloS One. 2013; 8: e53196 10.1371/journal.pone.0053196 23301042 PMC3534648

[55] Jiang Q, Wang F, Li MY, Ma J, Tan GF, Xiong AS. Selection of suitable reference genes for qPCR normalization under abiotic stresses in *Oenanthe javanica* (BI.) DC. PloS One. 2014; 9: e92262 10.1371/journal.pone.0092262 24651080 PMC3961309

[56] Jian B, Liu B, Bi Y, Hou W, Wu C, Han T. Validation of internal control for gene expression study in soybean by quantitative real-time PCR. BMC Mol Biol. 2008; 9: 59 10.1186/1471-2199-9-59 18573215 PMC2443375

[57] Chi X, Hu R, Yang Q, Zhang X, Pan L, Chen N, et al Validation of reference genes for gene expression studies in peanut by quantitative real-time RT-PCR. Mol Genet Genomics. 2012; 287: 167–176. 10.1007/s00438-011-0665-5 22203160

[58] Stajner N, Cregeen S, Javornik B. Evaluation of reference genes for RT-qPCR expression studies in hop (*Humulus lupulus* L.) during infection with vascular pathogen *Verticillium albo-atrum*. PloS One. 2013; 8: e68228 10.1371/journal.pone.0068228 23874551 PMC3709999

[59] Warzybok A, Migocka M. Reliable reference genes for normalization of gene expression in cucumber grown under different nitrogen nutrition. PloS One. 2013; 8: e72887 10.1371/journal.pone.0072887 24058446 PMC3772881

[60] Imai T, Ubi BE, Saito T, Moriguchi T. Evaluation of reference genes for accurate normalization of gene expression for real time-quantitative PCR in *Pyrus pyrifolia* using different tissue samples and seasonal conditions. PloS One. 2014; 9: e86492 10.1371/journal.pone.0086492 24466117 PMC3899261

[61] Ray DL, Johnson JC. Validation of reference genes for gene expression analysis in olive (*Olea europaea*) mesocarp tissue by quantitative real-time RT-PCR. BMC Res Notes. 2014; 7: 304 10.1186/1756-0500-7-304 24884716 PMC4062307

[62] Wang Y, Yu K, Poysa V, Shi C, Zhou Y. Selection of reference genes for normalization of qRT-PCR analysis of differentially expressed genes in soybean exposed to cadmium. Mol Biol Rep. 2012; 39: 1585–1594. 10.1007/s11033-011-0897-9 21625860

[63] de J Miranda V, Coelho RR, Viana AA, de Oliveira Neto OB, Carneiro RM, Rocha TL, et al Validation of reference genes aiming accurate normalization of qPCR data in soybean upon nematode parasitism and insect attack. BMC Res Notes. 2013; 6: 196 10.1186/1756-0500-6-196 23668315 PMC3660166

[64] Gu C, Chen S, Liu Z, Shan H, Luo H, Guan Z, et al Reference gene selection for quantitative real-time PCR in *Chrysanthemum* subjected to biotic and abiotic stress. Mol Biotechnol. 2011; 49: 192–197. 10.1007/s12033-011-9394-6 21416201

[65] Reddy PS, Rao TSRB, Sharma KK, Vadez V. Genome-wide identification and characterization of the aquaporin gene family in *Sorghum bicolor* (L.). Plant Gene. 2015; 1: 18–28.

