# Supplementary figures and images for: Identification and Validation of Reference Genes and Their Impact on Normalized Gene Expression Studies across Cultivated and Wild Cicer Species
Source: PLoS One. 2016 Feb 10;11(2):e0148451. doi: 10.1371/journal.pone.0148451 (PMC4749333; doi:10.1371/journal.pone.0148451)

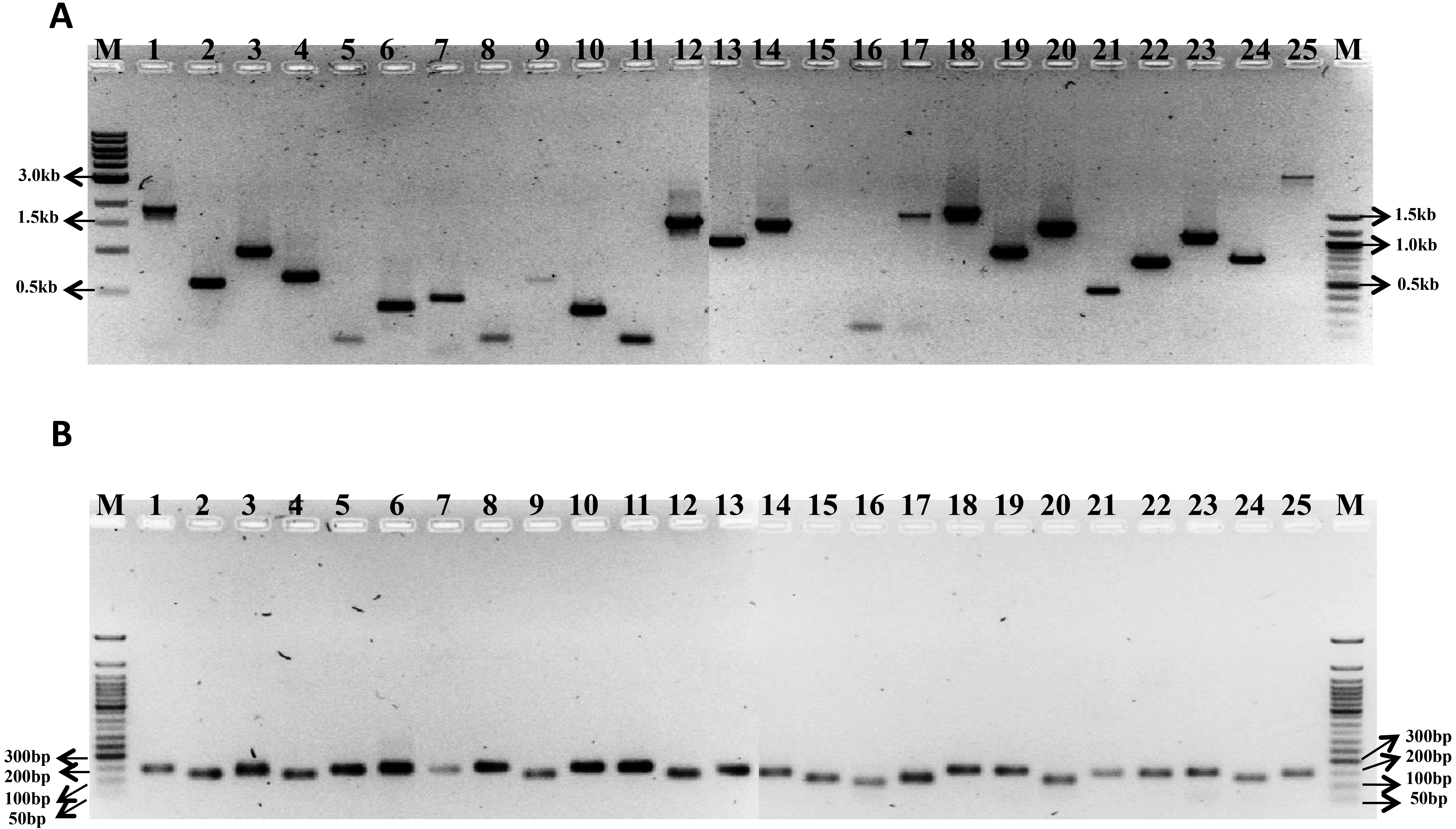

Supplement: S1 Fig — 1 to 25 indicates the loading order of the candidate reference genes as mentioned in Table 2, M- DNA size marker. All primer pairs except CYP, FBOX, HSP80 and PPR amplified a larger size PCR product with DNA template as compared to cDNA template, indicating the position of primer pairs spanning at least one intron. (TIF) [file pone.0148451.s001.tif]

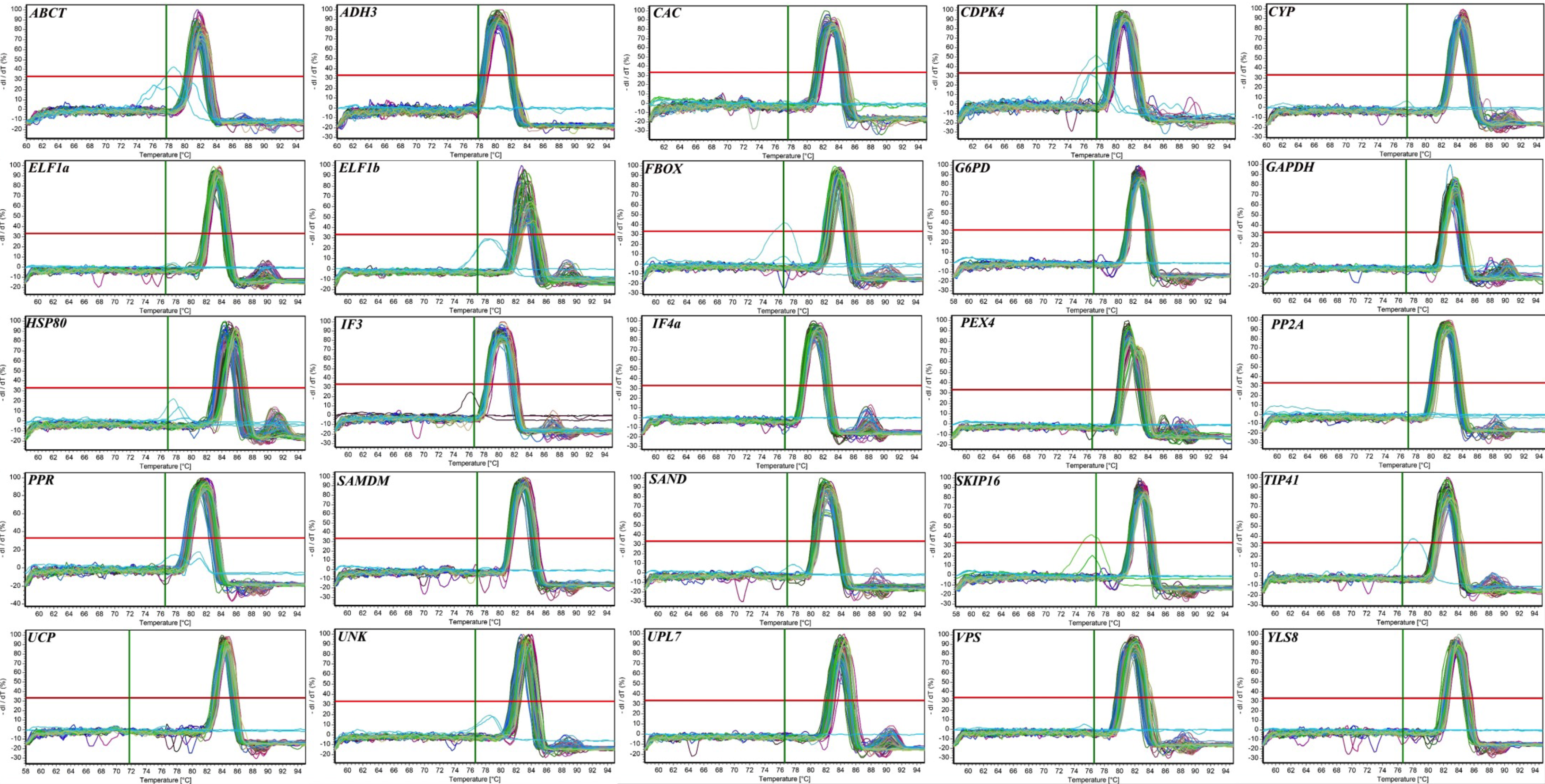

Supplement: S2 Fig — (TIF) [file pone.0148451.s002.tif]
